# Reflective seminars for the promotion of mental health in medical education: a qualitative model in a Latin American context

**DOI:** 10.3389/fpsyg.2026.1869011

**Published:** 2026-10-26

**Authors:** Raúl Sampieri-Cabrera, Diana P. Guizar-Sánchez, Adriana Robles-Cabrera, Tania Vives-Varela, Frida Contla-Cervantes

**Affiliations:** 1 Department of Physiology, Faculty of Medicine, National Autonomous University of Mexico, Mexico City, Mexico; 2 Department of Research in Medical Education, Secretariat of Medical Education, Faculty of Medicine, National Autonomous University of Mexico, Mexico City, Mexico

**Keywords:** health promotion, medical education undergraduate, medical students, mental health, psychological safety, qualitative research, reflective practice

## Abstract

**Background:**

Medical training sustains a persistent tension between academic demands, emotional well-being, and professional identity. In Latin America, mental health promotion remains scarcely integrated into the formal curriculum. This study examined a reflective seminar consisting of a single standardised four-hour session delivered once to each of 54 groups of second-year students (20 students per group; 1,080 students in total) during the preclinical phase of medical training. The study explored participants’ perceptions of the seminar’s pedagogical dynamics and its perceived effects.

**Methods:**

Qualitative interpretive research was conducted using a hermeneutic-phenomenological design. Four focus groups were held with 26 research participants (14 students and 12 teachers) who had taken part in the seminar and provided written informed consent. Inductive-deductive thematic analysis combined open and axial coding, source triangulation, and collegial calibration to support interpretive rigour.

**Results:**

Three interrelated dimensions were identified. Perceived institutional care legitimised well-being as an academic concern. Psychological safety favoured openness, peer support, and reflective deliberation. Teachers acted as emotional facilitators and models of professional vulnerability, which participants associated with a reconfiguration of pedagogical relationships and of the hidden curriculum. Neither psychological safety nor perceived benefit was uniform: some students remained disengaged, participation was lower in one group with a more reserved facilitator, and one teacher described feeling overwhelmed when students later sought individual support.

**Conclusion:**

The reflective seminar may function as a relational pedagogical infrastructure that articulates well-being and professional identity. Participants described curricular integration, a climate of trust, and teacher modelling as enabling ecological readings of distress and as displacing individualising narratives. These findings provide qualitative evidence of plausible, perceived mechanisms by which formative interventions can humanise medical education, and they generate hypotheses for comparable contexts rather than demonstrated effects.

## Introduction

University students’ mental health is a major concern of contemporary higher education (Herbert, 2022; Rockwell and Kimel, 2025), particularly in medical training, where demanding academic regimes, competitive institutional cultures, and barriers to timely psychosocial support (Brazeau et al., 2014; Rotenstein et al., 2016) coincide with a higher prevalence of depression, anxiety, exhaustion, and suicidal ideation than in other disciplines, pointing to structural rather than purely individual dynamics (Haykal et al., 2022; Tam et al., 2019; Quek et al., 2019).

Institutional responses have historically privileged reactive, individual-centred assistance activated after a crisis emerges (Bhat et al., 2023; Lea et al., 2026), with limited reach over structural determinants such as curricular organisation, the hidden curriculum, and faculty-student support (Lawrence et al., 2018; Parra Larrotta et al., 2025), prompting recent calls for preventive, population-level strategies embedded in the educational environment itself (Amini et al., 2025; Saad and Ali, 2025; West et al., 2016). This exposes a gap between abundant descriptive research on prevalence and risk factors and the scarcity of systematically evaluated curricular interventions (Ungar et al., 2022; Wadi et al., 2023), which matters because high distress coexists with low willingness to seek help, generating a cycle of silence (Bordbar et al., 2025; Hankir et al., 2020; Moutier, 2018).

Mental health problems during training also shape the construction of professional identity (Cotobal Rodeles et al., 2025; Monrouxe, 2010; Wald, 2015): values and models of professionalism are internalised during this period (Sarraf-Yazdi et al., 2021; Wald, 2015), and distress alters classroom dynamics and how students recognise themselves within the profession (De Rubeis et al., 2024; Dyrbye et al., 2020; Wang and Jiang, 2025). Faculty who reflexively share personal mental-health experiences have been shown to reduce stigma and foster dialogue, suggesting the value of interrelational strategies bringing student and faculty perspectives together (Branch et al., 2026; Loy and Kowalsky, 2024).

In Latin America this calls for locally situated analysis. Medical schools operate under structural inequality and pedagogical traditions that prioritise discipline over integral development (Hernández-Yasnó, 2023; Pacheco et al., 2017), and regional reviews converge on the scarcity of formally evaluated mental-health programmes and of faculty training in mental health literacy (Moon and D’Eon, 2024; West et al., 2016), constraining cultures of care and mutual support (L'hote et al., 2026; Wei et al., 2020). International proposals advocate integrating emotional well-being into the formal curriculum (Ferguson et al., 2022; Slavin et al., 2014): reflection spaces, socioemotional learning, and faculty mentoring show favourable effects on participation, stigma, and academic climate (Edmondson, 1999; McClintock et al., 2023), relocating mental health promotion to a constitutive component of medical professionalism (Altilio et al., 2022; Shapiro, 2023).

This study aligns with that perspective of curricular innovation. At the Faculty of Medicine of the National Autonomous University of Mexico, institutional indicators revealed high failure rates in the second year of the medical programme, associated with academic overload, emotional fatigue, and personal and socioeconomic tensions. In response, the Department of Physiology implemented a Reflective Seminar on Mental Health within the mandatory second-year curriculum, aimed at strengthening psychological protective factors through collective reflection, faculty–student dialogue, and explicit engagement with resilience, mentoring, motivation, and inclusive academic culture. This qualitative study did not assess whether the seminar was followed by a change in second-year failure rates; it focused on participants’ experiences and perceptions rather than on academic outcome indicators, and the question remains open for outcome-focused evaluation.

The study therefore addressed the following research question: how do students and teachers who participated in a curriculum-integrated reflective mental health seminar perceive its pedagogical dynamics, and how do they relate those perceptions to their well-being and to their professional identity formation?

This article reports a qualitative study of the experiences of students and faculty who participated in the seminar. From the analysis of their accounts, an interrelational model is proposed to show how the promotion of emotional well-being can be integrated into medical training. Situated in Latin America, the model is discussed in terms of its relevance and potential transferability to medical schools facing comparable cultural and institutional conditions. The study contributes to the literature on student well-being and professional identity by offering a perspective that moves away from deficit-based approaches toward an ecological, relational, and humanistic understanding of mental health in medical education.

## Methods

### Study design

Qualitative interpretive research was carried out with a hermeneutic-phenomenological design and elements of an educational case study, the implementation of a Reflective Seminar on Mental Health integrated into the physiology curriculum. The design allowed in-depth exploration of the perceptions and meanings attributed by the actors involved. A qualitative method was chosen because the interest lay in how participants experienced the seminar and what meaning they gave it in relation to their well-being and their training, questions that quantitative methods capture poorly.

The hermeneutic-phenomenological orientation (Gadamer, 2004; van Manen, 2016) was adopted because the aim was to interpret the meanings that students and teachers attributed to a shared experience, an approach widely used in health professions education research (Ajjawi and Higgs, 2007; Neubauer et al., 2019). An educational case-study lens (Stake, 1995; Yin, 2018) bounded the phenomenon to a single curriculum-integrated intervention in one institution, and this combined approach provided the overarching methodological and interpretive framework for the study. Analytic procedures were selected for their congruence with this orientation: thematic analysis (Braun and Clarke, 2006; Kiger and Varpio, 2020) combining inductive and deductive elements (Fereday and Muir-Cochrane, 2006), with open and axial coding used as flexible analytic techniques to organise and progressively relate meaning units and categories (Saldaña, 2021), and a “thinking with theory” strategy applied only after the empirically developed categories had been established (Jackson and Mazzei, 2012). Open and axial coding, although historically associated with grounded theory, were used only as analytic techniques within this interpretive orientation, not as components of a grounded-theory methodology, and “thinking with theory” served only to interpret categories already grounded in participants’ accounts; both are described in detail under Data analysis.

### Context and participants

The study took place in 2024 at the Faculty of Medicine of a Mexican public university and examined the implementation of a Reflective Seminar on Mental Health in the second year of physiology. The seminar was a single standardised four-hour face-to-face session, integrated into the regular schedule of laboratory practices and delivered once to each of the 54 groups into which the second-year cohort was divided across the study period; no group received a series of sequential sessions. Each group comprised 20 students, so the seminar reached the full second-year cohort of 1,080 students. Content addressed emotional regulation, resilience, academic stress, mentoring, professional identity, and institutional culture, articulated with students’ daily experience and with an explicit treatment of mental well-being as a formative dimension.

The seminar was a mandatory curricular activity delivered to the entire second-year cohort of 1,080 students as part of the regular physiology programme, and it would have been delivered in the same way irrespective of the research. Attending the seminar did not constitute participation in the research, and the 1,080 students who received it were not research participants by virtue of their attendance. No data were collected from the seminar sessions themselves for research purposes (the sessions were not recorded or observed by the research team). The research participants were exclusively the 26 individuals, 14 students and 12 teachers, who voluntarily took part in the focus groups after the seminar and provided written informed consent; their focus-group accounts constitute the only data analysed in this manuscript.

Each session was facilitated by one teacher, drawn from a pool of 12 facilitators who completed a four-hour preparatory orientation covering seminar objectives, group-facilitation guidelines, and referral protocols for students in distress. All groups worked from a common facilitator guide and shared materials: icebreaker activities, the “vocational lifeline” autobiographical narrative exercise, a brief resource-mapping activity identifying sources of support, guided small-group discussion, and a closing reflection. Adherence to the guide was not formally audited across groups, a point addressed in the Limitations section.

The qualitative evaluation used purposive (intentional) sampling by intensity, a strategy in which participants are deliberately selected because they are expected to offer information-rich accounts of the phenomenon under study rather than to be statistically representative. It brought together 26 research participants: 14 students and 12 teachers involved in facilitating the seminar, all of whom provided written informed consent to take part in the focus groups. Table 1 summarises participant demographics. Direct participation in the seminar gave students and teachers a shared experiential frame from which to comment on the pedagogical scope and the difficulties of the intervention.

**Table 1:** Participant demographics.

Characteristic	Students (*n* = 14)	Teachers (*n* = 12)
Mean age (years) SD	19 ± 1.7	32 ± 4.8
Gender distribution		
Women	7 (50%)	7 (58.3%)
Men	7 (50%)	5 (41.7%)
Mean teaching experience (years)	N/A	7 ± 6.8
Academic background	Second-year medical students	Predominantly postgraduate degree
Inclusion criterion	High seminar attendance and stated willingness to share experiences in depth	Served as seminar facilitators

### Recruitment

Invitations were issued at the end of the seminar and separately from curricular assessment, by personalised email clarifying that participation was voluntary, confidential, ungraded, and intended to improve the programme. Invitations made explicit that taking part in the research was distinct from the curricular seminar, which all students had already received, and that declining to participate would have no bearing on grades, academic standing, or any other aspect of the course. From the participating groups, a purposive sample of 14 students with high attendance and willingness to share experiences in depth was assembled. Teaching staff were contacted by email and in person at the end of the semester, and 12 facilitators took part; they were likewise informed that research participation was voluntary and independent of their teaching duties. Written informed consent was obtained from all 26 research participants before the focus groups.

Recruitment continued until information sufficiency was reached, operationalised as the point at which no new codes or units of meaning emerged during the calibration meeting following the fourth focus group, confirmed independently by two coders.

### Data collection

Focus groups were used because they capture how meaning is constructed collectively among participants who share an experience, which suited the collaborative format of the seminar and the sensitive content on emotions, vulnerability, and mental health, while also gathering multiple perspectives efficiently within single sessions. Four focus groups were conducted, two with students and two with teachers, to preserve homogeneity by role and favour free expression. Each session lasted approximately two and a half hours and was moderated by facilitators trained in qualitative methodology, external to the Department of Physiology and without direct teaching links to the participants, in order to reduce social desirability bias and support a climate of trust.

### Interview guides

A semi-structured guide was designed *ad hoc* through an iterative expert-panel review. The research team defined an initial set of topics from the literature and the objectives of the study and submitted it to an interdisciplinary panel of 6 medical education experts with at least 5 years of experience. Feedback was incorporated over two successive rounds until consensus was reached regarding thematic relevance and clarity of wording, operationally defined as a consensus threshold of ≥80% panel agreement.

The final guide covered seven analytical domains—content, didactic strategies, group dynamics, the role of the facilitator, student participation, perceived effects, and proposals for improvement—across twenty-four questions. Its structure remained equivalent for students and teachers, with semantic adjustments by role, which allowed shared thematic axes to be addressed from complementary positions. During sessions the guide operated flexibly, with probing questions guided by the discussion. All sessions were audio-recorded with informed consent and transcribed in full. Table 2 presents the *a priori* categories and sample questions. Transcripts were anonymised by assigning codes to participants (E1 for student 1, P2 for teacher 2, and so on) and removing references that could identify a person.

**Table 2:** *A priori* thematic areas and sample questions from the focus group guide.

Thematic area	Category focus	Sample guiding question
Seminar content	Relevance, perceived usefulness, additional or missing topics	“What do you think about the topics discussed (for example resilience, mentoring, vocation)? Would you add or remove any?”
Teaching strategies	Reflective modality, practical activities, materials used	“What did you think of the activities (narrative exercises, resource maps, and so on)? Which did you find most useful, and why?”
Group dynamics	Student–student and student–teacher interaction; emotional climate; time and space	“How would you describe the atmosphere during the sessions? Did you feel comfortable participating? What helped or hindered open discussion?”
Role of the teacher-facilitator	Facilitation style, closeness or authority, management of one’s own emotions	“(For teachers) Did you feel equipped to lead discussions about mental health? What difficulties and successes did you experience?”
Student attitudes	Level of engagement, openness to sharing, notable behaviours	“(For students) How did your classmates react to the seminar? Did you notice supportive attitudes or scepticism among your peers?”
Perceived effects	Personal and academic benefits; changes in attitude; awareness; criticisms	“Do you think the seminar had any effect on you (how you handle stress, how you see the career, your relationship with peers or teachers)? Why, or why not?”
Suggestions for improvement	Recommendations for the future, adjustments to format, frequency, curricular integration	“What would you change or add if the seminar were repeated next year? How could it be improved?”

### Data analysis

The corpus was analysed through thematic analysis (Braun and Clarke, 2006; Kiger and Varpio, 2020) supported by axial coding (Saldaña, 2021), integrated with a hermeneutic strategy and an inductive-deductive approach (Fereday and Muir-Cochrane, 2006). The analysis followed a structured, collaborative approach to thematic analysis that does not follow the reflexive variant (Braun and Clarke, 2021), in which explicitly defined coding procedures were combined with interpretive engagement with participants’ accounts. Working definitions and category boundaries were developed and iteratively refined by the analysts, without treating coding agreement as evidence of a single correct interpretation (Braun and Clarke, 2024; Kiger and Varpio, 2020). The deductive element consisted of the interview-guide domains, which sensitised the reading without fixing the final themes; codes, categories, and dimensions were developed inductively from the transcripts. Two team members read the transcripts independently and produced open coding to identify units of meaning, followed by axial coding used as a flexible second-cycle analytic technique to group conceptually related open codes into broader categories (Saldaña, 2021). Systematic calibration meetings refined the process by contrasting interpretations, examining divergent cases, and refining operational definitions, and documenting analytic decisions. Manual analytic work was supported by CAQDAS tools to maintain proximity to the empirical text. Identified categories were then compared across student and teacher groups to examine interpretive convergences and divergences across institutional positions. Only after the empirically developed axial categories had been established were they brought into dialogue with theoretical concepts through analytical memos, following the “thinking with theory” strategy (Jackson and Mazzei, 2012). Triangulation of sources and theory, collegial reflection, auditing of the coding process, and the use of representative quotations supported traceability and analytical transparency.

The progression from meaning units to final dimensions followed four steps: open coding of meaning units within each transcript; axial coding grouping related open codes into candidate categories; cross-case and cross-role comparison of these categories during calibration meetings; and interpretive integration of convergent categories, in dialogue with theoretical literature, into the three dimensions reported in Results. Table 3 illustrates this progression with representative examples.

**Table 3:** Progression from meaning units to codes, categories, and dimensions (illustrative examples).

Representative meaning unit	Open code	Axial category	Final dimension
“I feel like the department does care about us as people, not just as students.” (E1)	Affective recognition by the department	Institutional legitimation of well-being	1. Perceived institutional care
“We were all sitting at the same level… it felt different, not like a class.” (E8)	Horizontal spatial arrangement	Conditions enabling openness	2. Psychological safety and group dynamics
“To have the doctors tell us their stories, their mistakes, was striking.” (E6)	Faculty self-disclosure	Teacher as model of vulnerability	3. Teachers as emotional facilitators
“There were one or two who spent the session on their phones, as if it was beneath them.” (E3)	Persistent disengagement	Limits of group openness	2. Psychological safety and group dynamics

Axial coding originates in grounded theory, where it relates categories and subcategories through a coding paradigm in order to build substantive theory (Strauss and Corbin, 1990). In this study it was adapted following its description as a flexible second-cycle coding technique that can be used outside grounded theory (Saldaña, 2021) and was given one specific analytic function: to group open codes that shared meanings into broader axial categories, so that these could be compared across roles (students and teachers) and across focus groups. Grounded-theory procedures such as theoretical sampling, the coding paradigm, and the construction of a core category were not employed, and no substantive theory was pursued. Calibration meetings between the two analysts served as interpretive dialogue (comparing alternative readings, refining category boundaries, examining divergent cases, and documenting analytic decisions) rather than as a procedure to calculate intercoder reliability or to establish a single correct coding. The axial categories were then interpreted hermeneutically, moving iteratively between parts (codes and categories) and the whole (each transcript and the corpus) (Gadamer, 2004; Ajjawi and Higgs, 2007), until convergent categories were integrated into the three final dimensions. The interrelational model in Figure 1 is thus an interpretive, conceptual synthesis of these dimensions, not a grounded theory.

**Figure 1:**
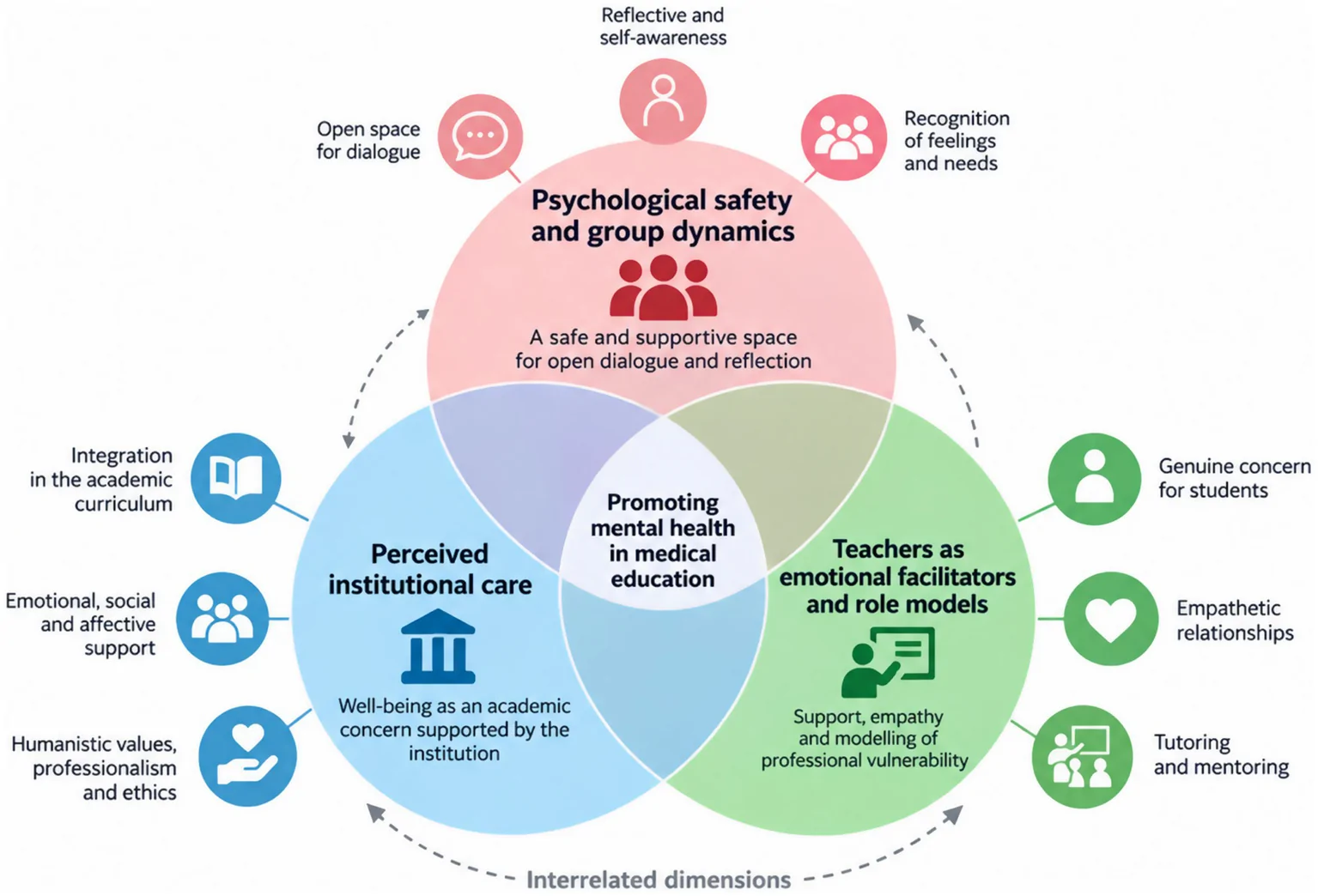
Interrelated dimensions of mental health promotion in medical education. The three dimensions identified (perceived institutional care, psychological safety and group dynamics, and teachers as emotional facilitators and role models) are depicted as non-hierarchically interrelated.

“Thinking with theory” (Jackson and Mazzei, 2012) was used only after the axial categories had been established and did not guide the coding. Through analytical memos, the categories were read in dialogue with theoretical concepts (psychological safety, hidden curriculum, habitus, performativity, and disciplinary power), asking what each concept made visible or problematic in participants’ accounts. This step deepened the interpretation of categories already grounded in the data and corresponds to the dialogue with theoretical literature in the fourth step of the progression described above; the resulting readings are developed in the Discussion. In this limited sense, the later interpretive phase was abductive in character: theoretically informed concepts were brought into iterative dialogue with empirically developed categories rather than used to predetermine the coding structure (Vila-Henninger et al., 2024; van Hulst and Visser, 2025).

For terminological consistency, the manuscript reserves “dimension” for the three overarching interpretive categories presented in Results (institutional care, psychological safety, and teacher facilitation); “code” and “axial category” refer to the intermediate analytic units shown in Table 3; and “thematic area,” used in Table 2, refers only to the *a priori* topics of the interview guide and should not be confused with the final dimensions.

### Reflexivity of the research team

Reflexivity was incorporated throughout, considering how institutional roles, disciplinary trajectories, and personal experiences shape the production and interpretation of data. The team combined researchers with internal knowledge of the context and members external to it, articulating situated understanding with analytical distance.

AR-C participated in the design and implementation of the seminar, acting as coordinator within the Department of Physiology; TV-V and FC-C moderated the four focus groups and had no prior teaching relationship with the participants; DG-S and RS-C led the formal coding and analysis. Pre-existing professional relationships between the coordinating researchers and some participating faculty members were documented as a potential source of social desirability bias and were explicitly addressed during calibration meetings.

Expectations were made explicit, interpretations discussed collegially, and the corpus analysed independently with segment rotation, in order to identify and mitigate confirmatory and social desirability biases. Interpretations were held to the empirical narratives and to their dialogue with the literature.

### Ethical considerations

The study was approved by the Faculty’s Ethics and Research Committee (FM/DI/120/2023). The Reflective Seminar on Mental Health was a curricular activity delivered to the full second-year cohort (1,080 students) as part of the regular programme and was not itself a research procedure; the research component consisted solely of the focus groups. Accordingly, the research participants were the 26 students and teachers (14 students and 12 teachers) who took part in the focus groups. All research participants gave written informed consent before data collection, after being informed of the aims of the study, the voluntary and ungraded nature of participation, their right to withdraw without consequences, the audio recording of the sessions, and the confidentiality measures adopted. Confidentiality, voluntary participation, and referral to institutional psychological support were ensured throughout, including a closing space at the end of each focus group. To protect confidentiality, participants were assigned alphanumeric codes (E for students, P for teachers), and names, specific professional roles, and other contextual details that could permit identification were removed from the transcripts. Only brief, de-identified quotations are reported in this article. Because the focus groups addressed sensitive mental-health experiences within a single medical school, and full transcripts could permit deductive identification even after anonymisation, the audio recordings and complete transcripts are accessible only to the research team and are not publicly shared (see Data availability statement).

## Results

Analysis identified three interrelated dimensions that frame the experiences students and teachers shared about the Reflective Seminar on Mental Health. The dimensions interact and together form a model of how the curricular intervention operates within the educational ecosystem; Figure 1 represents these relations schematically.

The results are organized by dimension, as each dimension constitutes, in essence, a phenomenon that transcends individual roles. Institutional support, psychological safety, and teaching facilitation were described from both student and teacher perspectives; part of the analytical interest lies in how these perspectives converge or diverge within a single dimension—an aspect upon which the interrelational model presented later depends. Within each dimension, the voices of students and teachers are identified separately using participant codes (E for students, P for teachers), allowing the origin of each unit of meaning to be traced. Table 3 shows how representative units of meaning were progressively grouped into open codes, axial categories, and the three final dimensions, presenting illustrative examples rather than the complete coding matrix.

### Dimension 1: perceived institutional care

The first dimension concerns how students interpreted the reflective seminar as a meaningful gesture by the institution, and specifically by the Department of Physiology, toward their emotional well-being. Many students found it surprising and encouraging that an academically demanding department allocated official teaching hours to emotions, self-care, and mental health. “I feel like the department does care about us as people, not just as students,” said one student (E1), voicing a widespread sense of recognition. Embedded in the formal curriculum, the seminar signalled that student mental health is a legitimate concern of medical education.

Students expressed appreciation for the initiative:

*“I would like to thank the Department of Physiology… it is one of those that demand the most from us academically, and it is one of the few, or perhaps the only department, that cares about our mental health. It teaches other departments that you can make space to support us…” (E4, student)*

In this account the seminar appears as a model other areas might follow, with the academic load set against a demonstration of care that the student marks as exceptional. Several participants described a symbolic shift from feeling that “only grades matter” to perceiving that they mattered as persons, and they valued protected time within their academic load: “Having that little time in the lab to talk about these issues… it was a respite, something we really needed” (E9). The preventive character of the intervention, integrating self-care into the routine instead of waiting for a crisis, was well received. The idea of “taking care of ourselves in order to take care of others” functioned in the discussions as a motto and carried a professional formative purpose: “They tell us we must be empathetic with patients, but how are we going to do it if we do not practise empathy with each other?” (E5).

Not every reaction was favourable at the outset. Some students initially read the intervention as cosmetic: “At first I thought it was just so they could say they do something for us, like for the photo” (E11). That perception shifted for most as sessions progressed, and students judged the authenticity of institutional care by how the sessions were run: horizontality, real listening, and follow-through on suggestions. Across the dimension, students described moving from feeling like numbers in an educational machine to feeling like subjects of care within their school, at least inside that microspace, with gratitude and a sense of belonging attached to that shift.

### Dimension 2: psychological safety and group dynamics

The second dimension identifies the socio-emotional climate of the seminar and the group dynamics as the conditions that made the reflective experience possible and, in some cases, limited it. Participants repeatedly stressed that sessions ran in an atmosphere of trust, respect, and openness, that is, with a high degree of psychological safety: the perception that one can show oneself as one is without fear of negative consequences for image, status, or career. It appeared in students’ willingness to share personal experiences, fears, or mistakes without expecting judgement or ridicule.

Several factors contributed to that atmosphere. The sessions abandoned the hierarchical format: they took place in the physiology laboratory, but the space was reorganised with chairs in a circle, outside the usual teacher-facing arrangement, with teachers seated as members of the circle. One student described it: “We were all sitting at the same level… it felt different, not like a class. The teachers told us we could speak freely, and so we did” (E8).

The participatory methodology also helped. Presentation games, small discussion groups, and creative activities worked as icebreakers and generated closeness: “When they had us do the vocational lifeline exercise, I ended up sharing things about my story that I had never talked about with my classmates… and seeing that they also shared, I felt supported” (E10). Shared vulnerability built trust, as some were encouraged to open up and others followed.

The facilitating role of teachers was a further condition of psychological safety, and students valued the active listening, empathy, and suspension of judgement most of them displayed: “The teacher assigned to our group said from the beginning that she would not be assessing us here, that she came to listen to us. And you could really see it; when someone spoke, she nodded, validated what they said, and shared something of her own” (E12). The account points to coherence between a facilitator’s discourse and practice.

Peer support formed an important subtheme. Students stressed that hearing classmates speak honestly about their struggles made them feel accompanied: “I realised I was not the only one who sometimes cannot handle everything any more” (E7). One student who had sought psychological help in the past shared this with the group, and a classmate who had been hesitant said it gave her the courage to consider seeking professional help. Structural conditions mattered as well, since students knew no assessment followed the session.

#### Divergent and less favourable experiences

The overall climate reported was positive, yet psychological safety was neither absolute nor homogeneous, and the heterogeneity deserves to be stated rather than smoothed into an idealised account. Some participants noted that not all peers were equally involved: “there were one or two who spent the session on their phones, as if it was beneath them, even judging us with their eyes” (E3). Resistance and cynicism persisted in a minority of students, plausibly reflecting lack of interest, personal discomfort with the topic, or scepticism toward the initiative. One student described being initially defensive and disengaged, becoming involved only after watching peers speak openly, which suggests that for part of the group psychological safety accumulated over the session rather than being available from the start.

In one teacher-facilitated group, a more reserved facilitation style coincided with comparatively lower student participation, which indicates that the seminar’s benefits were not uniform across the 54 groups and were sensitive to individual facilitator style, a source of variability discussed in Limitations. One student focus group also discussed how to handle sceptical peers: some proposed smaller sub-groups so that no one could hide behind the larger group, while others observed that disengaged classmates might need a kind of support the seminar could not offer. No participant reported acute emotional harm associated with the seminar. One teacher, however, described feeling overwhelmed when students later sought informal individual support beyond what he felt equipped to provide (see Dimension 3), which points to a boundary-related limit of the model rather than a uniformly positive effect.

### Dimension 3: teachers as emotional facilitators and role models

The third dimension addresses the experience and role of teachers: how their affective and pedagogical involvement shaped the dynamics, and what difficulties they faced in assuming an unconventional role. Teachers became more than content instructors, acting as emotional facilitators and as models of vulnerability and growth. Students perceived that teachers’ attitude shaped the climate of the sessions directly: “If the teacher showed confidence and talked about his own things, we all felt closer to him and talked more as well” (E13). The account suggests a modelling effect: teachers who shared their own experiences were read as authentic, which narrowed the distance from students and encouraged expression without fear.

One student commented, “To have the doctors tell us their stories, their mistakes, was striking… You always see them as super strong, and seeing that they went through the same thing encourages you a lot. They tell you: ‘Don’t worry, I could do it too and you can too’” (E6). A related reframing recurred across several students, who described moving away from an ideal of unconditional strength: “We are taught that we must always be strong, but in the seminar we saw that being strong also means knowing how to ask for help” (E14). Read together, these accounts indicate that by showing themselves vulnerable and resilient at once, teachers conveyed that feeling bad does not make one a bad doctor and that asking for help belongs to the formative path.

For participating teachers the experience took several forms. Several described discomfort or apprehension at having to address emotional issues with students, since they had not been trained for it: “At first I didn’t know if I was doing it well; I felt out of my comfort zone, because one is used to giving one’s lecture and that’s it” (P5). An orientation preceded the seminar, yet many still felt the weight of responsibility. One teacher described fearing an emotional overflow he would not know how to handle: “What if someone cried inconsolably or mentioned something serious, such as suicidal thoughts? I’m not a psychologist” (P7).

Afterwards, teachers recognised a wish to learn more about mentoring on this topic: “I realised how much I need to train myself in listening skills, in empathy… You think you are a good teacher just because you know your subject, but it goes beyond that” (P3). They also reported personal benefits, describing the seminar as a mirror of their own student years and as an occasion to rethink the teaching role: “I always thought my job was only to give knowledge; now I see I can, and should, also be a support for my students personally” (P9).

The transition carried tension. “So many years I got used to a certain distance, and suddenly being all the same was strange… but also beautiful. It was hard to take off my white coat, symbolically speaking, and just be me with them” (P2). Academic culture does not always look favourably on a teacher perceived as too close: one facilitator reported a colleague asking him why he was wasting his time, since “students need a firm hand, not cuddles” (P8).

Teachers also noticed changes in students’ behaviour toward them outside the seminar, describing students who now approached them with personal worries. This exposed the need for boundaries and resources, since one teacher felt overwhelmed when sought out and did not always know how to help beyond listening. No critical cases were reported during the sessions themselves; the post-seminar experience of being sought out for support beyond a facilitator’s competence nonetheless marks a boundary-related limitation of the model (see also Divergent and less favourable experiences, Dimension 2). Reflective seminars therefore belong within a broader ecosystem of support, with accessible mental health professionals and continuity of mentoring (see Table 4).

**Table 4:** Representative testimonies of students and teachers by dimension.

Dimension	Focus	Quotation (participant)
1. Perceived institutional care	Recognition and support from the department; symbolic and practical value of including mental health in the curriculum	“I really thank the Department of Physiology because… it’s one of the few, or perhaps the only department that cares about our mental health, and I think it teaches other departments that they can make space for us.” (E4, student) “At first I thought this was just to comply, but then I saw that they seriously took us into account. You feel that the faculty listens to you, that they do not just care about your grades.” (E11, student)
2. Psychological safety and group dynamics	Trust and openness among peers; participation without fear; sharing vulnerability; also obstacles, including sceptical attitudes	“The sessions were very good; there were times when I participated, and when I did, I felt heard, without shame… Knowing that many felt the same way I did gave me security.” (E7, student) “The only bad thing I saw that day was that one or two colleagues were being judgemental, on their phones… like they did not want to be there.” (E3, student) “We were giving each other courage. A classmate told us something very personal and no one laughed; on the contrary, we all respected it. That was good, you feel confident.” (E2, student)
3. Teachers as emotional facilitators	Authentic and close teaching role; teachers sharing experiences; effects on students and on teachers’ self-perception	“When doctors tell their stories you also feel that pressure of ‘they tell us: do not worry, you can do it’…” (E6, student) “At first it took me out of my comfort zone to be closer; one is not used to it. But seeing them open up… it made me remember why I wanted to be a teacher, to guide them not only in academic matters.” (P5, teacher) “Equipped to lead discussions on mental health? Not entirely, really. I had my doubts, but I learned along with them. This showed us areas where, as teachers, we must prepare ourselves more.” (P3, teacher)

These quotations articulate the central elements of each dimension: gratitude for institutional support (E4), emerging trust in group dynamics (E7) set against the resistance of a few (E3), and the influence of authentic teachers (E6) alongside teachers’ own reflexivity (P5, P3).

### Interrelational model

The three dimensions influence one another and converge in an interrelational model of mental health promotion in medical education. The findings suggest that for participants to experience such a curricular intervention as meaningful, it appears to require an explicit and tangible institutional commitment that legitimises the space for well-being (Dimension 1), safe learning environments in which students can express themselves and support one another (Dimension 2), and the active participation of prepared and engaged teachers who model emotional openness and sustain their own experience (Dimension 3). The absence of any of these is likely to weaken the perceived impact reported by participants. Without institutional backing the seminar risks remaining an isolated initiative; without a climate of trust students do not open up; without prepared teachers the activity can be derailed or trivialised.

The model operates in a dialectical logic and is not always harmonious. Tensions run between traditional and innovative institutional cultures, between participatory and sceptical students, and between teachers who remain in their comfort zone and those who explore new facets of their role. These tensions give the model a critical dimension and make visible that change involves confronting inertia and negotiating meanings. The seminar can be read as a space that opens a fissure in the dominant biomedical-academic order, through which alternative definitions of valid medical education become thinkable, and which comparative or mixed-methods research would need to test more directly. In that discourse, caring for students and encouraging them to care for one another belongs to the formation of good doctors rather than functioning as an add-on. Figure 1 represents these relations and the contextual factors that support or limit the intervention.

## Discussion

Participants’ accounts point to three interdependent processes: the institutional legitimation of care as an academic dimension; the reconfiguration of pedagogical relationships through deliberate practices of listening and faculty role modelling; and the opening of an interpretive repertoire that shifts individualising readings of distress toward relational and ecological frameworks (Vaughn and DeJonckheere, 2021; Wald, 2015). The literature on professional identity formation (PIF) describes professional identity as a situated construction emerging from the interaction between the formal curriculum, clinical practice, institutional narratives, and the tacit prescriptions of the hidden curriculum (Hafferty, 1998; Lamph et al., 2023; Ullah et al., 2025). Well-being and PIF share a common substrate, since both depend on how an institution defines excellence, vulnerability, help, error, and belonging (Pinkasová and Fialová, 2026; Saad and Ali, 2025). The data indicate that guided reflexivity operates as a bridge between them, enabling a language of personal agency and collective responsibility that reorders normative expectations about the “good doctor” (De La Fuente et al., 2022; Quaintance et al., 2025; Sandars and Patel, 2020). The phrase “caring for ourselves in order to care for others” works as an emic synthesis of that shift, with self-care emerging as an ethical condition of clinical practice (Epstein, 2020; Monrouxe, 2010; Shanafelt et al., 2019).

The repeated reframing of the question “what kind of doctor do I want to be” suggests explicit identity work activated by the seminar (Sarraf-Yazdi et al., 2021, 2024). In international studies, exposure to physicians’ narratives about mental health and vulnerability is associated with stigma reduction and with an expansion of the boundaries of acceptable professionalism (Heley et al., 2020; Meeks et al., 2020). The present results converge with that evidence, since faculty modelling of legitimate vulnerability opens a path for students to integrate technical competence with humanity, personal limits, and help-seeking (Bird et al., 2020; Kumagai, 2014).

Theoretically, the pattern resonates with narrative approaches to PIF that conceive identity as a plot rewritten through shared stories, particularly when institutions provide interpretive frames for naming tensions between ideals and lived experience (Charon, 2006; Palla et al., 2024). As reported in Results (Dimension 3), the seminar temporarily disrupted teachers’ accustomed professional distance, described by one teacher as needing to “take off my white coat” in order to relate to students on a more horizontal footing. This can be read as a partial and temporary reconfiguration of the conventional performance of the medical-teacher role, in which an alternative and more relational way of enacting that role becomes provisionally available (cf. Butler, 1990, on the performative and revisable character of social roles). Some teachers experienced the reconfiguration as refreshing and others as disorienting, which underlines that identity work of this kind is neither automatic nor uniformly comfortable.

The quotation “being strong also means knowing how to ask for help”, presented in Results (Dimension 3), condenses an identity reframing with ethical implications. Research on moral distress shows that the pressure to always appear strong and high-performing can wear down trainees’ sense of integrity and belonging, with knock-on effects on empathy, learning, and mental health (Bordbar et al., 2025; Cotobal Rodeles et al., 2025). By treating moments of struggle as a normal and expected part of training rather than as personal failure, the seminar appears to give students a place to name these difficulties and to see asking for help as an option, which may reduce guilt and widen the range of responses they feel able to make to their own distress and that of others. This is consistent with the view that well-being forms part of professional competence, since it underlies clinical judgement, communication, patient safety, and the capacity to sustain a long career (Epstein, 2020; Shanafelt et al., 2019).

Literature on psychological safety in health sciences education (Edmondson, 1999; McClintock et al., 2023) indicates that deep learning requires relational conditions that permit questioning, error, uncertainty, and moral deliberation. Here, curricular integration of the seminar sent an institutional signal of legitimacy: mental health moved from a peripheral position to an academic dimension with protected time. Participants interpreted that institutional gesture as a plausible mechanism contributing to the “perceived care” reported in the focus groups, translating declared values into concrete practices within official schedules.

The same legitimation invites a more critical reading. It is worth asking whether an institutional gesture of this kind, valuable as it is, could also operate as a way of normalising certain emotions within limits that remain acceptable to the institution, in the sense discussed by Foucault (1995) in relation to disciplinary power. The present data cannot settle the question, since only participants’ own accounts were available. Most testimonies pointed toward feeling empowered and validated rather than surveilled, which suggests that, at least as experienced by these participants, the space functioned more as recognition than as control. Research combining participant accounts with independent observation would be needed to examine the tension directly.

The device also acts on the hidden curriculum (Martimianakis et al., 2015). Multiple studies report that tacit messages of toughness, hyperindividual competition, and emotional silencing shape climates that foster burnout and cynicism (Maity et al., 2025; Passi et al., 2013). The intervention introduces explicit counter-messages: asking for help acquires the status of strength, vulnerability is read as a human experience compatible with excellence, and mutual support is framed as future clinical competence (Nielsen et al., 2024). This pedagogical micro-politics aligns with approaches that intervene in the hidden curriculum through repeatable institutional practices rather than normative declarations (Ansari et al., 2026).

Faculty role modelling emerges as a central element. International evidence indicates that role modelling exerts sustained influence on PIF when teachers make their ethical reasoning and their relationship with personal limits visible (Bransen et al., 2024). In the present data, exposure to faculty who share personal experiences related to mental health appears to expand what can be said, with effects on stigma and belonging (Moutier, 2018). As reported in Results (Dimension 2), students described this modelling as temporarily suspending the customary hierarchical distance between teacher and student. Read through Bourdieu’s (1990) notion of habitus, facilitators can be seen as provisionally embodying an alternative, more sympathetic disposition alongside their usual authority-bearing habitus, which several students linked explicitly to their own willingness to speak. The mechanism suggests that the seminar may function as an infrastructure of trust provided that pedagogical design preserves authenticity, confidentiality, and clear role boundaries. Literature on mentoring and socioemotional learning notes that faculty self-disclosure requires ethical guidelines and institutional preparation to prevent boundary ambiguity, emotional overload, or therapeutic expectations (Martin et al., 2020). The formative effect observed therefore signals both the potential and the need for pedagogical governance.

That need was explicit in teachers’ own accounts. Several facilitators described uncertainty about how to respond if a student disclosed suicidal ideation or another situation exceeding their competence, and one teacher reported feeling overwhelmed when students later sought informal individual support (see Results, Dimensions 2 and 3). Fostering emotional openness in an educational setting cannot rest on facilitator goodwill alone. It requires clear referral protocols to institutional mental health services, an explicit and communicated delimitation of the teacher-facilitator role as distinct from that of a mental health professional, targeted training in psychological first aid and risk recognition, and timely access to qualified professionals for students and teachers alike. Without these safeguards, scaling an intervention of this type could expose facilitators to situations beyond their preparation and expose students to inconsistent responses to genuine risk; with them, the model becomes more transferable to other institutions.

The results also support an interpretive shift from deficit-centred explanations toward ecological, relational, and organisational models of well-being. The faculty statement that many of the problems have to do with the fact that “we are not creating the conditions for that pressure to be manageable” expresses a reattribution toward shared institutional responsibility, aligned with ecological frameworks that propose multilevel interventions spanning individual skills, interpersonal networks, teaching practices, school policies, and assessment norms (O’Reilly et al., 2018; Slavin et al., 2014). The burnout literature indicates that isolated interventions focused on individual resilience show limited effects when structural burdens, punitive assessment, or climates of extreme competition persist. According to participants’ accounts, the seminar appears to act at the individual and interpersonal levels, with a symbolic institutional component that legitimises the topic; whether it also produces measurable structural or longitudinal change remains to be tested (see Limitations).

The rhetoric of well-being can also function as a productivity device through which institutions promote self-care to enhance performance, a tension visible in debates that frame wellness as a governing technique individualising structural problems (Song and Baicker, 2019). The present findings allow an alternative reading, since participants interpreted care as an institutional gesture of recognition and legitimation of shared vulnerability.

## Limitations

The interpretation rests on focus groups in a single institutional setting, so the conclusions describe plausible and conceptually transferable mechanisms with limited inferential scope. The absence of comparators and of repeated measurement makes it impossible to estimate changes attributable to the seminar in climate, perceived well-being, academic trajectories, or PIF, particularly across clinical transitions when the hidden curriculum tends to intensify. Scaling would require implementation evaluation with frameworks such as RE-AIM or CFIR, and validated measures of psychological safety, moral distress, and burnout that permit comparison with international literature and examination of heterogeneity by subgroup.

Four further limitations should be noted. Purposive sampling favoured students with high seminar attendance and stated willingness to share experiences in depth; this criterion probably selected participants with more favourable dispositions toward the intervention and may have inflated positive appraisals of institutional care, group climate, and faculty engagement, so the findings reflect the experience of engaged participants rather than the full range of student responses, including those of students who attended irregularly or disengaged. Social desirability bias cannot be ruled out despite external moderation of the focus groups, particularly because some members of the research team held pre-existing professional relationships with teacher-participants and coordinated the seminar itself; this dual role, addressed in the Reflexivity section, may have shaped both what participants chose to share and how the team interpreted it. The 54 groups that received the seminar were not audited for fidelity of delivery, so variability in facilitation style and content emphasis across groups, partly illustrated by the divergent experiences reported in Results, may have influenced participants’ experiences in ways this study cannot quantify. No longitudinal follow-up was conducted, so the durability of perceived effects on well-being, group climate, or professional identity beyond the seminar remains unknown.

## Conclusion

The reflective seminar integrated into the curriculum may act, according to participants’ accounts, as a pedagogical infrastructure articulating well-being and identity formation, by transforming educational relationships, legitimising vulnerability as a component of professionalism, and enabling ecological readings of distress. The main contribution lies in identifying, from qualitative evidence, plausible and perceived mechanisms by which a relational intervention may recalibrate the hidden curriculum and open paths toward a more empathetic academic culture. Given the exploratory, single-institution, and cross-sectional design, these mechanisms should be read as hypotheses for future and more systematically evaluated implementations rather than as demonstrated effects. Transferability further depends on the safeguards discussed above: explicit referral protocols, a clearly delimited teacher-facilitator role, and preparation in psychological first aid for those who facilitate.

## Data Availability

The datasets presented in this article are not readily available because they consist of focus-group audio recordings and transcripts containing sensitive information about participants’ mental-health experiences within a single medical school. Even after removal of names, the complete transcripts contain contextual details, personal experiences, professional roles, and quotations that could permit deductive identification of participants. In line with the confidentiality commitments made to participants in the informed consent process and with the conditions of approval granted by the Ethics and Research Committee of the Faculty of Medicine, UNAM (FM/DI/120/2023), the raw transcripts are not publicly shared. De-identified illustrative excerpts supporting the findings are reported in the article (Tables 3, 4). Requests to access further de-identified material (e.g., the coding framework or additional anonymised excerpts) should be directed to the corresponding author, DG-S dguizar@facmed.unam.mx; any such access will be subject to approval by the Ethics and Research Committee and to the confidentiality conditions agreed with participants.
